# SARS-CoV-2 productively infects human gut enterocytes

**DOI:** 10.1126/science.abc1669

**Published:** 2020-05-01

**Authors:** Mart M. Lamers, Joep Beumer, Jelte van der Vaart, Kèvin Knoops, Jens Puschhof, Tim I. Breugem, Raimond B. G. Ravelli, J. Paul van Schayck, Anna Z. Mykytyn, Hans Q. Duimel, Elly van Donselaar, Samra Riesebosch, Helma J. H. Kuijpers, Debby Schippers, Willine J. van de Wetering, Miranda de Graaf, Marion Koopmans, Edwin Cuppen, Peter J. Peters, Bart L. Haagmans, Hans Clevers

**Affiliations:** 1 Viroscience Department, Erasmus Medical Center, Rotterdam, Netherlands.; 2 Oncode Institute, Hubrecht Institute, Royal Netherlands Academy of Arts and Sciences and University Medical Center, Utrecht, Netherlands.; 3 The Maastricht Multimodal Molecular Imaging Institute, Maastricht University, Maastricht, Netherlands.; 4 Center for Molecular Medicine and Oncode Institute, University Medical Centre Utrecht, Utrecht, Netherlands.; 5 Hartwig Medical Foundation, Amsterdam, Netherlands.

## Abstract

The virus severe acute respiratory syndrome–coronavirus 2 (SARS-CoV-2) can cause coronavirus disease 2019 (COVID-19), an influenza-like disease that is primarily thought to infect the lungs with transmission via the respiratory route. However, clinical evidence suggests that the intestine may present another viral target organ. Indeed, the SARS-CoV-2 receptor angiotensin converting enzyme 2 (ACE2) is highly expressed on differentiated enterocytes. In human small intestinal organoids (hSIOs), enterocytes were readily infected by SARS-CoV and SARS-CoV-2 as demonstrated by confocal- and electron-microscopy. Consequently, significant titers of infectious viral particles were detected. mRNA expression analysis revealed strong induction of a generic viral response program. Hence, intestinal epithelium supports SARS-CoV-2 replication, and hSIOs serve as an experimental model for coronavirus infection and biology

Severe acute respiratory syndrome (SARS), caused by coronavirus SARS-CoV, emerged in 2003 (*1*). In late 2019, a novel transmissible coronavirus (SARS-CoV-2) was noted to cause an influenza-like disease ranging from mild respiratory symptoms to severe lung injury, multi-organ failure, and death (*2*–*4*). SARS-CoV and SARS-CoV-2 belong to the *Sarbecovirus* subgenus (genus *Betacoronavirus*, family *Coronaviridae*) (*5*–*7*). The SARS-CoV receptor is the angiotensin-converting enzyme 2 (ACE2) (*8*, *9*). The spike proteins of both viruses bind to ACE2, whereas soluble ACE2 blocks infection by SARS-CoV as well as by SARS-CoV-2 (*10*–*13*). Transmission of SARS-CoV-2 is thought to occur through respiratory droplets and fomites. The virus can be detected in upper respiratory tract samples, implicating the nasopharynx as site of replication. In human lung, ACE2 is expressed mainly in alveolar epithelial type II cells and ciliated cells (*14*–*16*). However, highest expression of ACE2 in the human body occurs in the brush border of intestinal enterocytes (*14*, *17*). Even though respiratory symptoms dominate the clinical presentation of COVID-19, gastrointestinal symptoms are observed in a subset of patients (*18*, *19*). Moreover, viral RNA can be found in rectal swabs, even after nasopharyngeal testing has turned negative, implying gastro-intestinal infection and a fecal–oral transmission route (*20*–*22*).

## SARS-CoV-2 infects airway and gut organoids

**Fig. 1:**
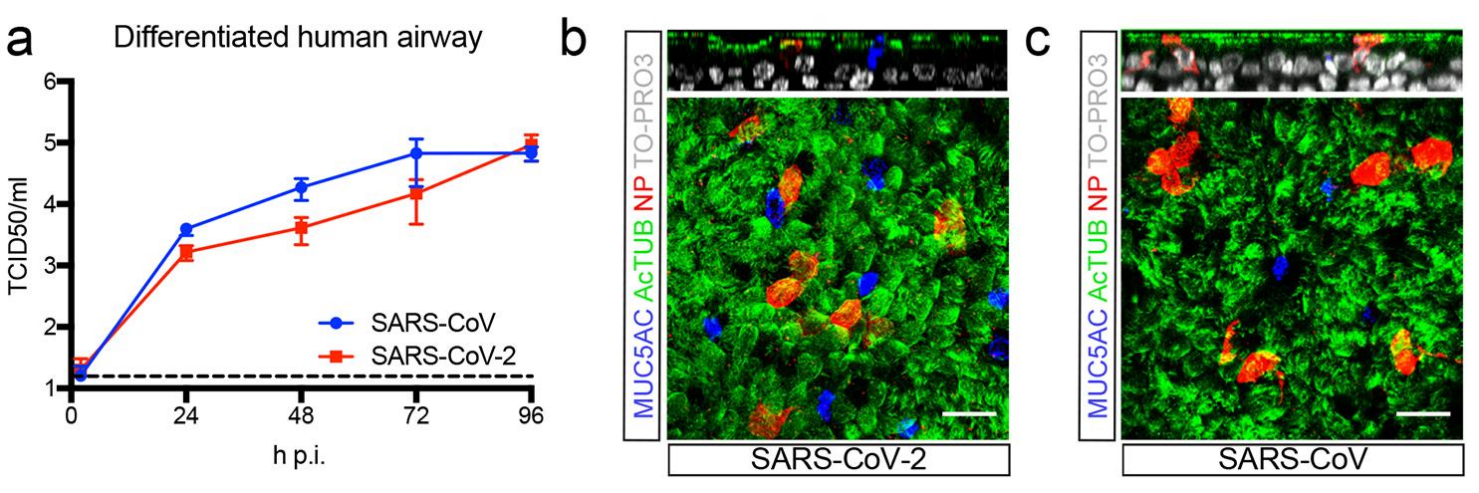
SARS-CoV and SARS-CoV-2 infect 2D human airway cultures. (**a**) Live virus titers can be observed by virus titrations on VeroE6 cells of apical washes at 2, 24, 48, 72 and 96h after infection with SARS-CoV (blue) and SARS-CoV-2 (red). The dotted line indicates the lower limit of detection. Error bars represent SEM. N=4. *P<0.05, **P<0.01, ***P<0.001. (**b** and **c**) Immunofluorescent staining of SARS-CoV-2 (b) and SARS-CoV (c) infected differentiated airway cultures. Nucleoprotein (NP) stains viral nucleocapsid (red), which colocalized with the ciliated cell marker AcTUB (green). Goblet cells are identified by MUC5AC (blue). Nuclei are stained with TO-PRO3 (white). Scale bars indicate 20μM. Top panels are side-view while bottom panels are top-view.

Organoids are 3D structures, that can be grown from adult stem cells (ASCs) and recapitulate key aspects of the organ from which the ASCs derive. Since SARS-CoV and SARS-CoV-2 target the lung, we added virus to organoid-derived human airway epithelium cultured in 2D and observed that SARS-CoV and SARS-CoV2 readily infected differentiated airway cultures. (Fig. 1A). Immunostaining reveal that the viruses targeted ciliated cells, but not goblet cells (Fig. 1, B and C).

**Fig. 2:**
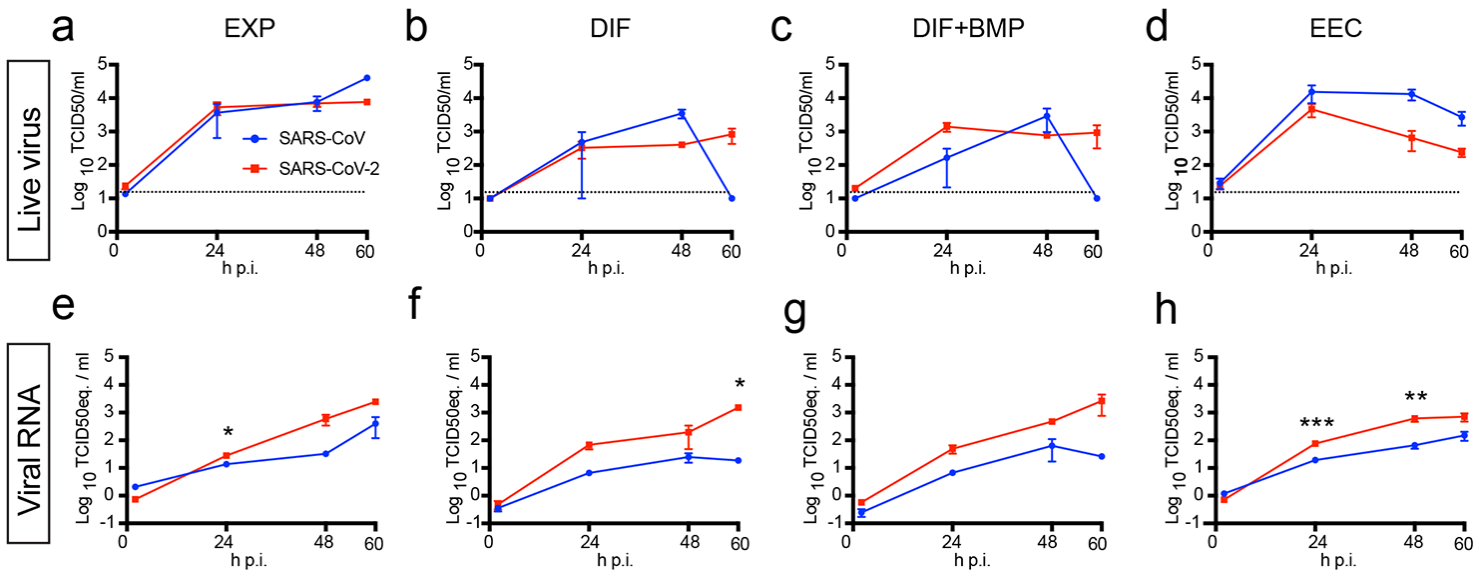
SARS-CoV and SARS-CoV-2 replicate in hSIOs. (**a** to **d**) Live virus titers can be observed by virus titrations on VeroE6 cells of lysed organoids at 2, 24, 48 and 60h after infection with SARS-CoV (blue) and SARS-CoV-2 (red). Different medium compositions show similar results. (**e** to **h**) qPCR analysis targeting the E gene of similar timepoints and medium compositions as (a) to (d). The dotted line indicates the lower limit of detection. Error bars represent SEM. N=3. *P<0.05, **P<0.01, ***P<0.001.

**Fig. 3:**
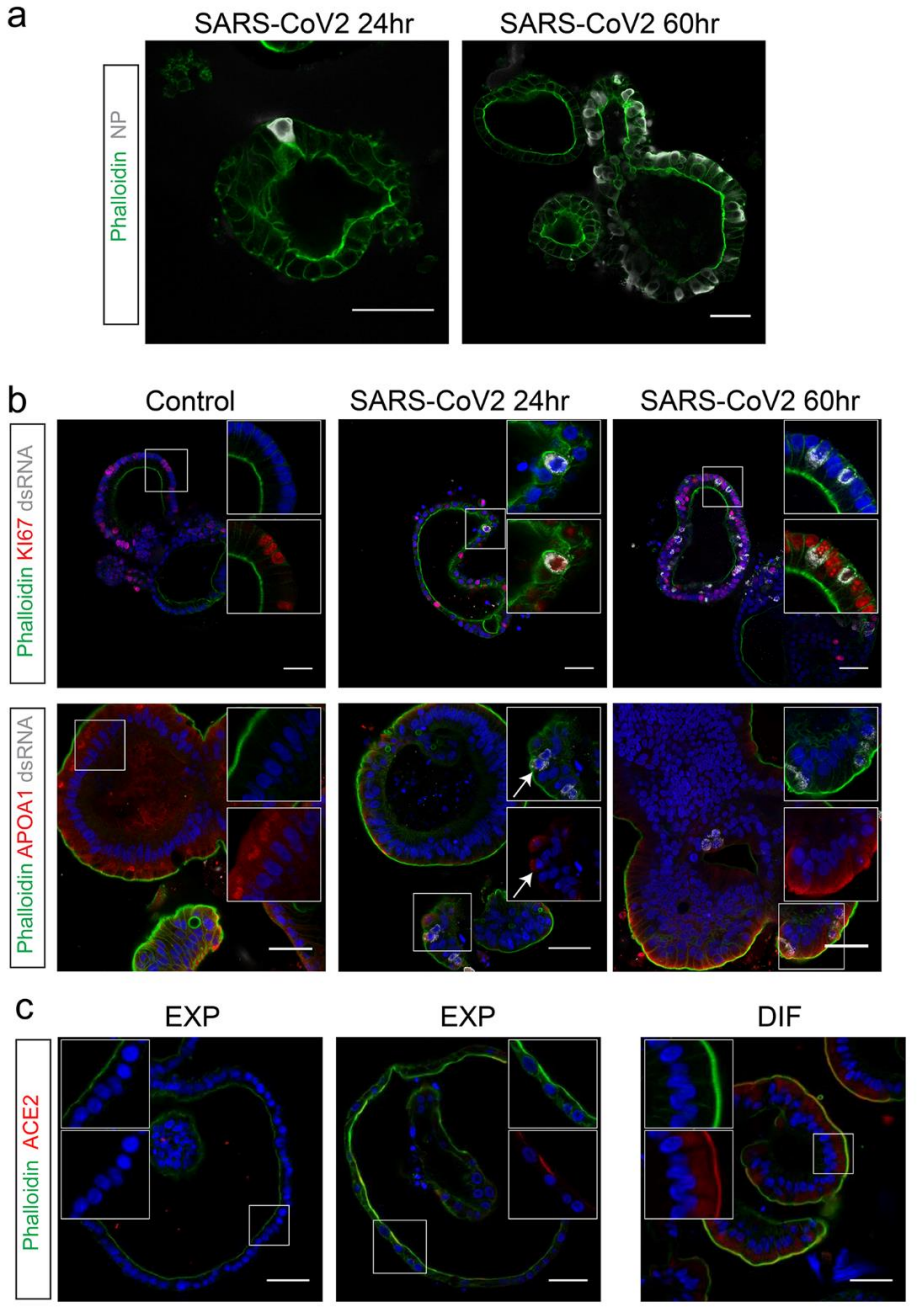
SARS-CoV-2 infects proliferating cells and enterocytes. (**a**) Immunofluorescent staining of SARS-CoV-2-infected intestinal organoids. Nucleoprotein (NP) stains viral capsid. After 24 hours, single virus-infected cells are generally observed in organoids. These small infection clusters spread through the whole organoid after 60 hours. (**b**) SARS-CoV-2 infects both post-mitotic enterocytes identified by Apolipoprotein A1 (APOA1) and dividing cells that are KI67-positive. Infected cells are visualized by dsRNA staining. Enterocytes are shown in differentiated organoids, and proliferating cells in expanding organoids. Arrows point to APOA1-positive cells. (**c**) Immunofluorescent staining of ACE2 in intestinal organoids in expansion and differentiation condition. All scale bars are 50 μm.

**Fig. 4:**
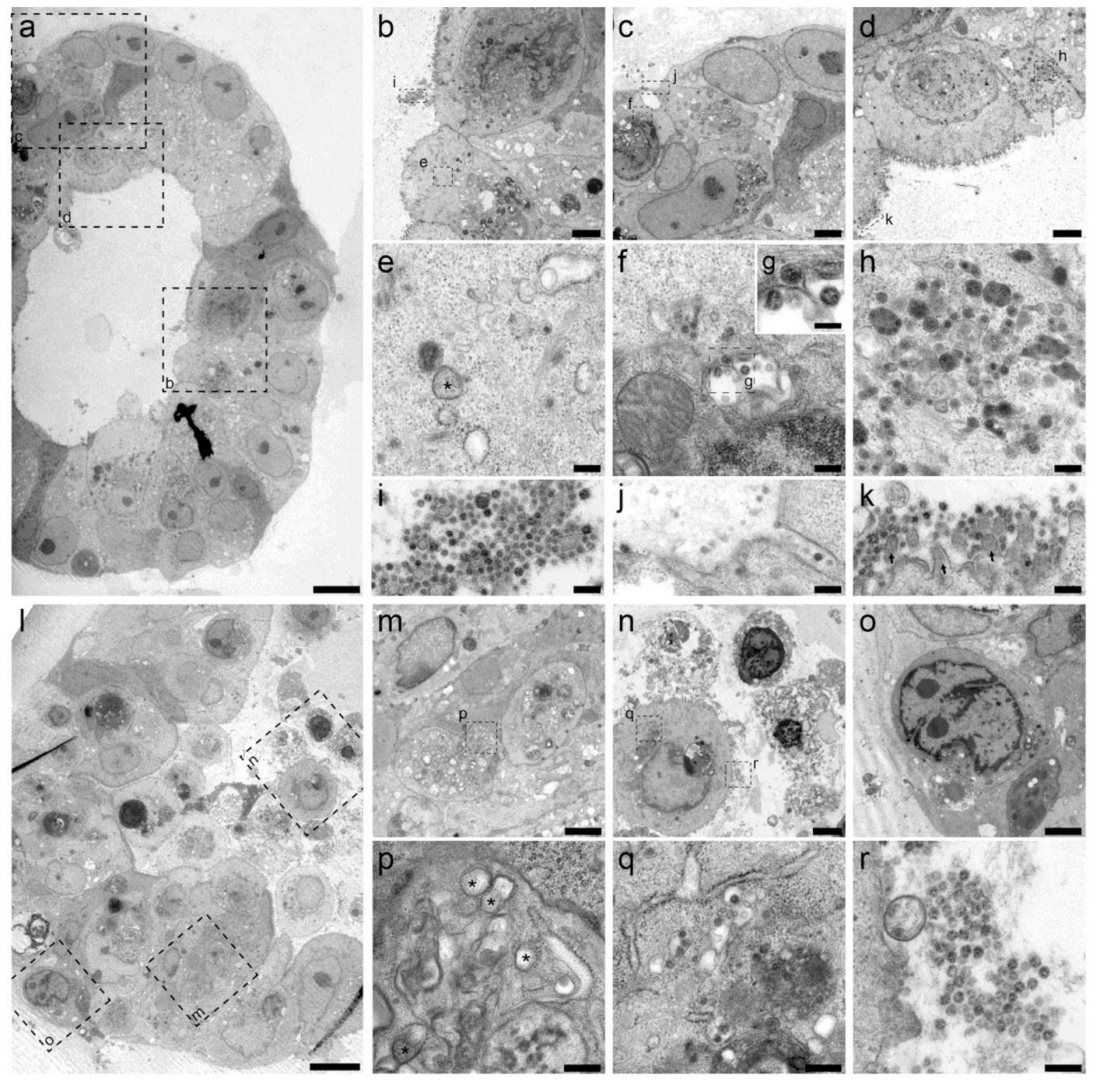
Transmission electron microscopy analysis of SARS-CoV-2 infected intestinal organoids. (**a** to **h**) Overview of an intact organoid (a) showing the onset of virus infection (b to d) at different stages of the viral lifecycle, i.e., early double membrane vesicles (DMVs) [(e), asterisk], initial viral production in the Golgi apparatus [(f) and (g)] and complete occupation of virus particles inside the endomembrane system (h). (**i** to **k**) Extracellular viruses are observed in the lumen of the organoid (i), and are found at the basal (j) and apical side (k) alongside the microvilli (arrows). Scale bars represent 10 μm (a), 2.5 μm [(b) to (d)], 250 nm [(e), (f), and (h) to (k)] and 100 nm (g). (**l** to **q**) Overview of an organoid (l) showing severely infected cells [(m) and (o)], disintegrated cells (o) and stressed cells as evident from the atypical nucleoli (p). Intact cells reveal DMV areas of viral replication [(p), asterisks] and infected Golgi apparatus (q). (**r**) Extracellular clusters of viruses. Scale bars represent 10 μm (l), 2.5 μm [(m) to (p)] and 250 nm [(p) to (r)]. Data was deposited to the Image Data Resource (https://idr.openmicroscopy.org) under accession number idr0083.

**Fig. 5:**
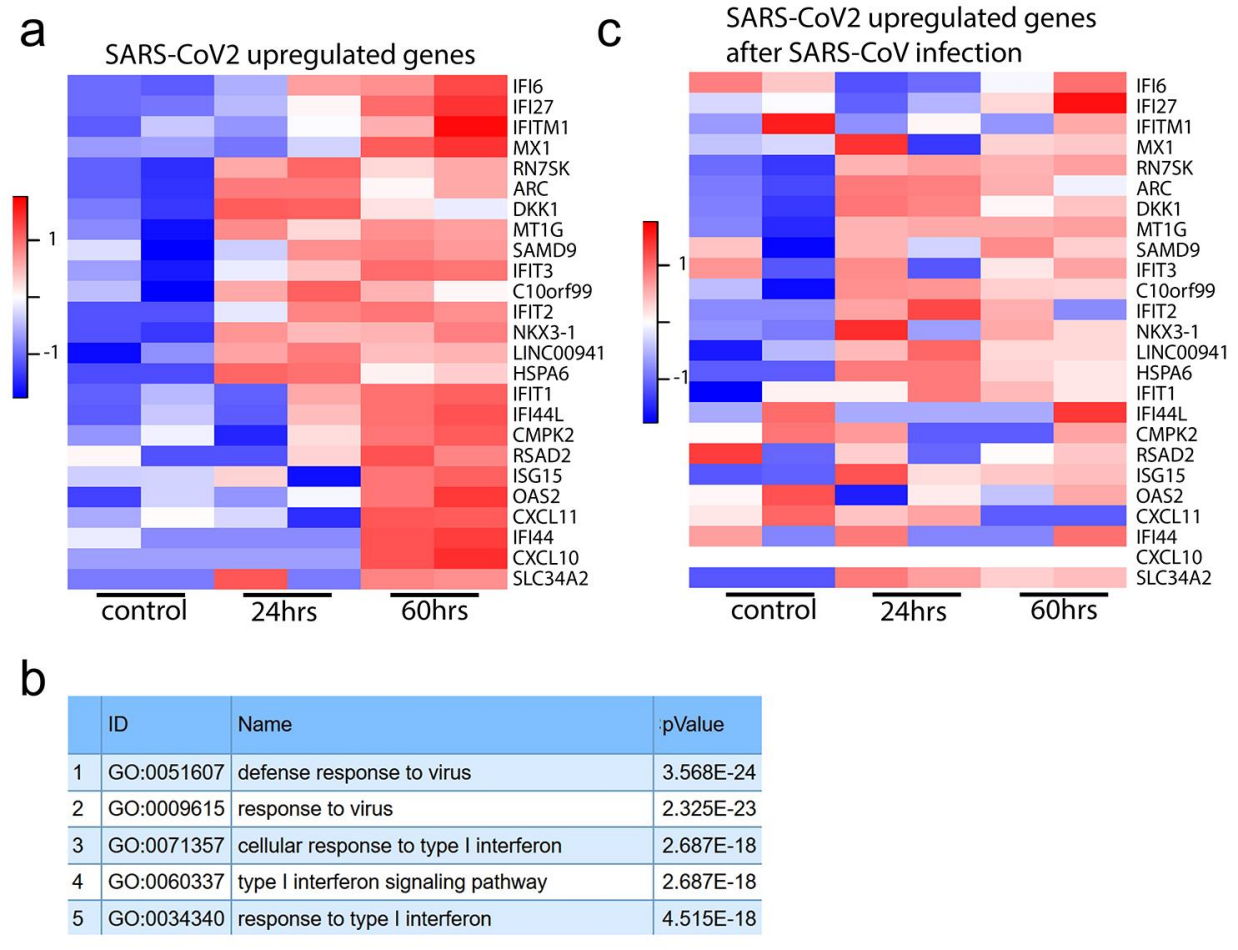
Transcriptomic analysis of SARS-CoV-2 infected intestinal organoids. (**a**) Heatmaps depicting the 25 most significantly enriched genes upon SARS-CoV-2 infection in expanding intestinal organoids. (**b**) Colored bar represents Z-score of log2 transformed values. GO term enrichment analysis for biological processes of the 50 most significantly up-regulated genes upon SARS-CoV-2 infected in intestinal organoids. (**c**) Heatmaps depicting the genes from (a) in SARS-CoV infected expanding organoids. Colored bar represents Z-score of log2 transformed values.

Human small intestinal organoids (hSIOs) are established from primary gut epithelial stem cells, can be expanded indefinitely in 3D culture and contain all proliferative and differentiated cell types of the in vivo epithelium (*23*). Of note, hSIOs have allowed the first in vitro culturing of Norovirus (*24*). We exposed ileal hSIOs grown under four different culture conditions (‘EXP’, ‘DIF’, ‘DIF-BMP’ and ‘EEC’) to SARS-CoV and SARS-CoV-2 at a multiplicity of infection (MOI) of 1. hSIOs grown in Wnt-high expansion medium (EXP) overwhelmingly consist of stem cells and enterocyte progenitors. Organoids grown in differentiation medium (DIF) contain enterocytes, goblet cells, and low numbers of enteroendocrine cells (EECs). Addition of BMP2/4 to DIF (DIF-BMP) leads to further maturation (*25*). In the final condition, we induced expression of NeuroG3 from a stably transfected vector with doxycycline to raise EECs numbers (fig. S3D). Samples were harvested at multiple timepoints post infection and processed for the analyses given in Figs. 2 to 5. Both SARS-CoV and SARS-CoV-2 productively infected hSIOs as assessed by qRT-PCR for viral sequences and by live virus titrations on VeroE6 cells (see Fig. 2 for lysed organoids and fig. S1 for organoid supernatant). Infectious virus particles and viral RNA increased to significant titers for both viruses in all conditions. Since EXP medium supported virus replication (Fig. 2, A and E), enterocyte progenitors appeared to be a primary viral target. Differentiated organoids (DIF; DIF-BMP) produced slightly (non-statistically significant) lower levels of infectious virus (Fig. 2 and fig. S1). In organoids induced to generate EECs, virus yields were similar to those in EXP medium (Fig. 2, D and H). In differentiated hSIOs, SARS-CoV-2 titers remained stable at 60 hours post infection, whereas SARS-CoV titers dropped 1-2 log (Fig. 2, B, C, F, and G). The latter decline was not observed in infected hSIOs grown in EXP. Culture supernatants across culture conditions contained lower levels of infectious virus compared to lysed hSIOs, implying that virus was primarily secreted apically (fig. S1, A to D). Despite this, viral RNA was detected readily in culture supernatants correlating with the infectious virus levels within hSIOs (Fig. 2, E to H, and fig. S1, E to H).

ACE2 mRNA expression differed greatly between the four conditions. EXP-hSIOs express 300-fold less ACE2 mRNA compared to DIF-hSIOs, when analyzed in bulk (fig. S2). BMP treatment induced 6.5-fold up-regulation of ACE2 mRNA compared to DIF treatment alone. Since this did not yield infection rate differences, the DIF-BMP condition was not analyzed further.

## SARS-CoV-2 infects enterocyte lineage cells

To determine the target cell type, we then performed confocal analysis on hSIOs cultured in EXP, DIF, or EEC conditions. We stained for viral dsRNA, viral nucleocapsid protein, KI67 to visualize proliferative cells, actin (using phalloidin) to visualize enterocyte brush borders, DNA (DAPI) and cleaved caspase 3 to visualize apoptotic cells. Generally, comparable rates of viral infections were observed in the organoids growing in all three conditions. We typically noted staining for viral components (white) in rare, single cells at 24 hours. At 60 hours, the number of infected cells had dramatically increased (Fig. 3A). Infected cells invariably displayed proliferative enterocyte progenitor-phenotypes (EXP; Fig. 3B, top) or ApoA1+ enterocyte-phenotypes (DIF; Fig. 3B, bottom). Of note, SARS-CoV also readily infected enterocyte lineage cells (fig. S3, A and B) as shown previously (*26*, *27*). Some infected enterocyte progenitors were in mitosis (fig. S3C). Whereas EEC-organoids produced appreciable titers, we never observed infection of Chromogranin-A^+^ EECs (fig. S3, D and E). We also did not notice infection of Goblet cells across culture conditions. At 60 hours, apoptosis became prominent in both SARS-CoV and SARS-CoV-2 infected enterocytes (fig. S5). ACE2 protein was readily revealed as a bright and ubiquitous brush border marker in hSIOs in DIF medium (Fig. 3C). In hSIOs in EXP medium, ACE2 staining was much lower -yet still apical- in occasional cells in a subset of organoids that displayed a more mature morphology (Fig. 3C). In immature (cystic) organoids within the same cultures, the ACE2 signal was below the detection threshold. The percentage of infected organoids under EXP and DIF conditions are given in fig. S4. Figure S5 shows images and quantification of apoptotic cells upon infection.

## Ultrastructural analysis of the viral life cycle in enterocytes

Unsupervised transmission electron microscopy (TEM) (*28*) was performed on selected highly infected samples. Figure 4 shows two hSIOs, selected from 42 imaged hSIOs at 60 hours post SARS-CoV-2 infection. These differ in the state of infection: whereas the cellular organization within organoid 1 was still intact (Fig. 4A, entire organoid; B to D, intermediate magnification; E to K, high magnification), many disintegrated cells can be seen in organoid 2 (Fig. 4, bottom; L, entire organoid; M to O, intermediate magnification; P to R, high magnification). Viral particles of 80-120 nm occurred in the lumen of the organoid (Fig. 4I), at the basolateral (Fig. 4J) and apical side (Fig. 4K) of enterocytes. The double-membrane vesicles which are the subcellular site of viral replication (*29*) are visualized in Fig. 4, E and P. The nuclei in both organoids differed from nuclei in mock-infected organoids by a slightly rounder shape. The nuclear contour index (*30*) was 4.0+/−0.5 vs 4.3+/−0.5 for control set. There was more heterochromatin (4N), and one or two dense nucleoli in the center (4O).

## RNA expression changes in infected enterocytes

We then performed mRNA sequence analysis to determine gene expression changes induced by SARS-CoV and SARS-CoV-2-infection of hSIOs cultured continuously in EXP medium and hSIOs cultured in DIF medium. Infection with SARS-CoV-2 elicited a broad signature of cytokines and interferon stimulated genes (ISGs) attributed to type I and III interferon responses (Fig. 5A and tables S1 and S2), as confirmed by Gene Ontology analysis (Fig. 5B). An overlapping list of genes appeared in SARS-CoV-2-infected DIF organoids (fig. S6 and table S3). RNA sequencing analysis confirmed differentiation of DIF organoids into multiple intestinal lineages, including ACE2 up-regulation (fig. S7). SARS-CoV also induced ISGs, yet to a much lower level (table S4). Figure 5C visualizes the regulation of SARS-CoV-2-induced genes in SARS-CoV infected organoids. This induction was similar to infections with other viruses like norovirus (*31*), rotavirus (*32*) and enteroviruses (*33*, *34*). A recent study (*35*) describes an antiviral signature induced in human cell lines after SARS-CoV-2 infection. Whereas the ISG response is broader in intestinal organoids, the induced gene sets are in close agreement between the two datasets (fig. S8). One striking similarity was the low expression of Type I and III interferons: we only noticed a small induction of the Type III interferon IFNL1 in SARS-CoV-2 infected organoids. In SARS-CoV-infected organoids, we did not observe any type I or type III interferon induction. We confirmed these findings by ELISA on culture supernatant and qRT-PCR, which in addition to IFNL1 picked up low levels of type I interferon IFNB1 in SARS-CoV-2 but not in SARS-CoV infected organoids (fig. S9). The specific induction of IP-10/CXCL10 and ISG15 by SARS-CoV-2 was also confirmed by ELISA and qRT-PCR, respectively (fig. S10). As in (*35*), a short list of cytokine genes was induced by both viruses albeit it to modest levels. For a comparison with (*35*), see fig. S11. Altogether these data indicate that SARS-CoV-2 induces a stronger interferon response than SARS-CoV in HIOs.

Finally, the infection was repeated in a second experiment in the same ileal HIO line and analyzed after 72 hours. Analysis involved viral titration (fig. S12), confocal imaging (fig. S13), and RNA sequencing (fig. S14). This experiment essentially confirmed the observations presented above. A limited, qualitative experiment applying confocal analysis demonstrated infectability of two other lines available in the lab (one ileal, one duodenal) from independent donors (fig. S13). This study shows that SARS-CoV and SARS-CoV-2 infect enterocyte lineage cells in a human intestinal organoid model. We observed similar infection rates of enterocyte-precursors and enterocytes whereas ACE2 expression increases ~1000-fold upon differentiation at the mRNA level (fig. S2). This suggests that low levels of ACE2 may be sufficient for viral entry.

SARS-CoV-2 is the third highly pathogenic coronavirus (after SARS-CoV and MERS-CoV) to jump to humans within less than 20 years suggesting that novel zoonotic coronavirus spillovers are likely to occur in the future. Despite this, limited information is available on coronavirus pathogenesis and transmission. This is in part due to the lack of in vitro cell models that accurately model host tissues. Very recently, it was shown that human iPS cells differentiated toward a kidney fate support replication of SARS-CoV-2 (*13*). Our data imply that human organoids represent faithful experimental models to study the biology of coronaviruses.

## Supplementary Materials

science.sciencemag.org/cgi/content/full/science.abc1669/DC1 Materials and Methods Figs. S1 to S14 Tables S1 to S4 References (*36*–*44*) MDAR Reproducibility Checklist
